# Comparison of arch forms between Turkish and North American

**DOI:** 10.1590/2177-6709.21.2.051-058.oar

**Published:** 2016

**Authors:** Ahmet A. Celebi, Hakan Keklik, Enes Tan, Faruk I. Ucar

**Affiliations:** 1 Assistant professor, Zirve University, Department of Orthodontics, School of Dentistry, Gaziantep, Turkey.; 2 Postgraduate student, Kirikkale University, Department of Orthodontics, School of Dentistry, Kirikkale, Turkey.; 3 Assistant professor, Kirikkale University, Department of Orthodontics, School of Dentistry, Kirikkale, Turkey.; 4 Assistant professor, Selcuk University, Department of Orthodontics, School of Dentistry, Konya, Turkey.

**Keywords:** Dental arch, Dental model, Ethnic groups, Malocclusion

## Abstract

**Objective:**

The aim of this study was to clarify the morphological differences in the mandibular arches of Turkish and North American white subjects.

**Methods:**

The sample included 132 Turkish (34 Class I, 58 Class II, and 40 Class III) and 160 North American (60 Class I, 50 Class II, and 50 Class III) subjects. The most facial portion of 13 proximal contact areas was digitized from photocopied images of patients' mandibular dental arches. Clinical bracket points were calculated for each tooth based on mandibular tooth thickness data. Four linear and two proportional measurements were taken. The subjects were grouped according to arch form types (tapered, ovoid and square) in order to have frequency distribution compared between ethnic groups in each Angle classification.

**Results:**

The Turkish group showed significantly lower molar depth and more significant molar width-depth (W/D) ratio in all three Angle classifications. On the other hand, the Turkish group also showed a significantly larger intercanine width in Class III malocclusion and intermolar width in Class II malocclusion. The most frequent arch forms seen were the ovoid arch form in the Turkish group and the tapered form in the white group.

**Conclusions:**

Our results demonstrate that when treating Turkish patients, one should expect to use preformed ovoid arch form orthodontic wires in a significant percentage of patients.

## RESUMO

**Objetivo:**

o objetivo deste estudo foi avaliar as diferenças morfológicas entre as arcadas dentárias inferiores de indivíduos turcos e indivíduos norte-americanos leucodermas.

Métodos: a amostra incluiu 132 indivíduos turcos (34 Classe I, 58 Classe II e 40 Classe III) e 160 norte-americanos (60 Classe I, 50 Classe II e 50 Classe III). A porção mais vestibular de 13 áreas de contato proximal foi digitalizada a partir de imagens impressas das arcadas dentárias inferiores dos pacientes. Os pontos de colagem dos braquetes foram calculados, para cada dente, com base em dados referentes à espessura dos dentes inferiores. Quatro medidas lineares e duas proporcionais foram obtidas. Os indivíduos foram classificados de acordo com o formato da arcada (triangular, oval e quadrada), permitindo que a frequência de cada formato fosse comparada entre os grupos étnicos, e dentro de cada classificação de Angle.

**Resultados:**

o grupo composto por indivíduos turcos apresentou profundidade da arcada na região dos molares significativamente menor, além de uma proporção largura/profundidade da arcada na região dos molares maior em todas as classificações de Angle. Em contrapartida, esse mesmo grupo mostrou maiores distâncias intercaninos nos pacientes Classe III e intermolares nos pacientes Classe II. A arcada oval foi a mais frequente no grupo composto por indivíduos turcos, enquanto a arcada triangular foi a mais frequente no grupo composto por indivíduos norte-americanos leucodermas.

**Conclusões:**

os resultados obtidos mostram que, ao tratar pacientes turcos, o profissional deve prever o uso de arcos ortodônticos pré-conformados ovais em um percentual significativo dos pacientes.

## INTRODUCTION

The dental arch, fundamental principle in orthodontic planning and therapy, is an important element in Orthodontics.^1^ Therefore, correct identification of a patient's arch form is a crucial parameter in achieving a stable, functional and esthetic orthodontic treatment result, since failure to preserve the arch form might increase the probability of relapse.^2^ ^,3^

Over the years, human dental arch form has been recognized to be variable in shape and size. It is described by many authors in geometric forms (ellipse, parabolic curve and hyperbolic) and mathematical functions.^4^ A number of studies have used normal, untreated samples to determine arch form mathematically^5^ ^,6^ or to characterize arch form through various measurements, with incisal edges and cusp tips as landmarks.^7^ ^,^ ^8^ ^,^ ^9^

Classic studies have described that well-aligned dental arches are roughly categorized as square, ovoid, and tapered.^10^ These arch forms can also be expressed as narrow, normal and wide.^11^ Especially when determining the arch wire forms to be used at the initial phase of treatment, Chuck^12^ advocated that making a choice between these three forms would be better than using a single arch form. Due to this cause, the most convenient arch form type, according to patient's ethnical origin and malocclusion, should be chosen for preformed superelastic arch wires in leveling and arrangement phases.^13^ ^,14^

The dolichocephalic head form is the most common among North American whites. The Turkish population, however, originates from heterogeneous ethnic backgrounds: Asiatic Turks, Kurds, the Balkans, Caucasus, Middle East, Iran as well as from ancient Romans, Byzantines, and Arabs; also, Turkey, is an Eurasian country located in Western Asia (mostly in the Anatolian peninsula) and in Southeastern Europe (East Thracian).^15^ Therefore, head form of the Turkish might well differ from the white population.

Studies on the arch forms of the Turkish and comparisons with other ethnic groups have not been performed previously. The aim of this study was to determine the morphological differences between Turkish and North American white clinical mandibular arches in Class I, Class II, and Class III malocclusions by measuring patients' arch dimensions. The subjects were grouped according to arch form (tapered, ovoid and square) in order to have the frequency distribution of the three arch forms clarified for comparison between the ethnic groups in each Angle classification.

## MATERIAL AND METHODS

This study was based on two sample groups of Turkish and North American white subjects. The Turkish group consisted of pretreatment mandibular dental models from 34 Class I, 58 Class II, and 40 Class III patients obtained from the Kirikkale University Department of Orthodontics, Turkey. The North American white group consisted of models from 60 Class I, 50 Class II, and 50 Class III patients from the University of Southern California, Department of Orthodontics, Los Angeles, and a private practice in San Diego, California, USA (Table 1). 

**Table 1:** Sex and age comparisons between North American and Turkish samples.

		North American	Turkish	*p* value
		(n = 160)	(n = 132)	
Total % (n)	Male	47.5 (76)	43.9 (58)	0.54 NS
	Female	52.5 (84)	56.1 (74)	
Class I % (n)	Male	38.3 (23)	35.3 (12)	0.77 NS
	Female	61.7 (37)	64.7 (22)	
Class II % (n)	Male	52.0 (26)	51.7 (30)	0.97 NS
	Female	48.0 (24)	48.3 (28)	
Class III % (n)	Male	54.0 (27)	40.0 (16)	0.18 NS
	Female	46.0 (23)	60.0 (24)	
Age (years) Mean (SD)	Total	15.4 (5.2)	13.9 (2.5)	
	Class I	16.6 (5.9)	13.8 (2.5)	
	Class II	14.7 (4.7)	14.4 (1.9)	
Class III	14.5 (4.3)	13.4 (3.2)	

*p* > 0.05, NS = non-significant, Chi-square test.

The samples were selected to match the following criteria: (1) Angle dental Class I, II, and III malocclusions; (2) permanent dentition with normal tooth size and shape; (3) no supernumerary teeth; (4) arch-length discrepancy of 3 mm or less; (5) absence of restorations extending to contact areas, cusp tips, or incisal edges; and (6) no previous orthodontic treatment.

**Figure 1:**
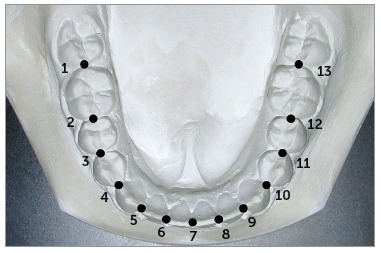
Points digitized on the occlusal photocopy. These points represent the most facial portions of 13 proximal contact areas. Modified from: Nojima et al.^14^, 2001.

The occlusal surfaces of the mandibular models were photocopied, with a ruler included for magnification correction. The photocopied images were placed on a digitizer, and the most facial portions of 13 proximal contact areas around the arch were digitized (Fig 1). These points are used to estimate corresponding bracket slot locations (clinical bracket point) for each tooth. The proximal contact between the two central incisors was used as the origin of the x-y coordinate.

The original x-y coordinate on the digitizer was corrected for magnification and adjusted to establish a new x-y coordinate, so that the mean inclination of straight lines connecting the right and left contact points between the first and second premolars as well as those between the second premolars and first molar became parallel to the original x-axis.

The perpendicular to a line connecting mesial and distal contact points of each tooth on the coordinate was drawn from the midpoint of the mesiodistal line for incisors, canines, and premolars and from the mesial third of the line for molars. The perpendicular was extended labially or buccally to locate a clinical bracket point for each tooth, according to mandibular teeth thickness data of Andrews.^16^

**Figure 2:**
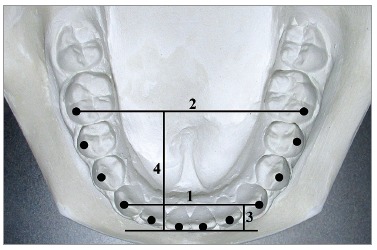
Twelve clinical bracket points, four linear and two proportional measurements of arch dimensions: 1) intercanine width; 2) intermolar width; 3) canine depth; 4) molar depth; 5) canine W/D ratio; and 6) molar WD ratio. Modified from: Nojima et al.^14^, 2001.

The following four linear and two proportional measurements were made (Fig 2): 

1) Intercanine width: the distance between canine clinical bracket points.
2) Intermolar width: the distance between first molar clinical bracket points.
3) Canine depth: the shortest distance from a line connecting the canine clinical bracket points to the origin between central incisors.
4) Molar depth: the shortest distance from a line connecting the first molar clinical bracket points to the origin between central incisors.
5) Canine width-depth (W/D) ratio: the ratio of intercanine width and canine depth.
6) Molar W/D ratio: the ratio of intermolar width and molar depth.

In addition, 12 clinical bracket points were printed per patient at full size to select, from square, ovoid and tapered arch forms (OrthoForm; 3M Unitek, Monrovia, California, USA), the arch form that best fits the eight clinical bracket points from first premolar to first premolar (Fig 2).

## STATISTICAL ANALYSIS

Power analysis showed that, for this study, α = 0.01; β = 0.20 (1-β = 0.80; power = 0.8225); and power of 82 % were needed, so as to detect a difference of 1 mm. Power analysis showed that 32 patients were required in each group. Statistical evaluation was performed with SPSS 16.0 software (SPSS Inc., Chicago, IL, USA). Chi-square test was used to assess the association between sex and the two ethnic groups: North American and Turkish groups. The association between arch form and ethnic group was also assessed by means of chi-square test. Analysis of variance (ANOVA) was performed to compare the adjusted means of arch dimensions between the two ethnic groups by Angle classification and arch form separately. The results of the continuous variables were compared by ANOVA (for three subgroups) or by two-sample t-test for differences in means (for two subgroups). All analyses were tested at a significance level of 0.05. 

## RESULTS

Measurement errors were assessed by statistically analyzing the difference between duplicate measurements taken at least two weeks apart on 24 casts selected at random. Measurement errors were generally small (less than 5% of the measured mean value) and within acceptable limits. 

Tables 2 and ^3^ depict the arch dimension measurements and results of the t-test for the North American and Turkish Class I, II, and III samples. The Turkish group showed significantly smaller molar depth and bigger molar W/D ratio in all three Angle classifications. In addition, the Turkish group also showed a significantly larger intercanine width in Class III malocclusion and intermolar width in Class II malocclusion. When Class I, II, and III malocclusions were combined, statistically significant differences were observed in canine depth, molar depth, canine W/D ratio and molar W/D ratio between the two ethnic groups.

**Table 2:** Complete sample comparison between North American white and Turkish groups.

Variable	White (n = 160) Mean (SD)	Turkish (n = 132) Mean (SD)	p value
Intercanine width (mm)	29.07 (1.39)	29.19 (1.67)	> 0.05
Intermolar width (mm)	49.42 (2.61)	49.77 (2.71)	> 0.05
Canine depth (mm)	6.26 (1.13)	5.84 (1.00)	< 0.05*
Molar depth (mm)	27.08 (2.07)	25.37 (1.87)	< 0.01**
Canine W/D ratio	4.79 (0.85)	4.92 (0.77)	< 0.05*
Molar W/D ratio	1.84 (0.17)	1.97 (0.17)	< 0.05*

*p < 0.05; **p < 0.01, Two-sample t-test for difference in means.

**Table 3:** Comparison between North American and Turkish groups by Angle classification. Values presented as: Mean (SD).

Variable	Class I (n = 94)		p value	Class II (n = 108)		p value	Class III (n = 90)		p value
	White (n = 60)	Turkish (n = 34 )		White (n = 50)	Turkish (n = 58)		White (n = 50)	Turkish (n = 40)	
Intercanine width (mm)	29.01 (1.26)	28.96 (1.36)	> 0.05	28.92 (1.22)	28.87 (1.88)	> 0.05	29.29 (1.68)	29.74 (1.56)	< 0.05*
Intermolar width (mm)	49.17 (2.29)	48.97 (2.29)	> 0.05	48.5 (2.53)	49.25 (2.24)	< 0.05*	50.62 (2.65)	49.75 (3.46)	> 0.05
Canine depth (mm)	6.3 (0.88)	6.33 (0.92)	> 0.05	6.79 (1.12)	6.85 (0.81)	> 0.05	5.69 (1.15)	3.95 (0.94)	< 0.01**
Molar depth (mm)	26.84 (1.62)	25.63 (1.64)	< 0.05*	27.3 (2.12)	26.03 (1.80)	< 0.05*	27.02 (2.59)	24.20 (1.63)	< 0.05*
Canine W/D ratio	4.68 (0.56)	4.59 (0.62)	> 0.05	4.37 (0.7)	4.35 (0.50)	> 0.05	5.34 (1.0)	6,02 (0.91)	< 0.01**
Molar W/D ratio	1.84 (0.11)	1.91 (0.14)	< 0.05*	1.78 (0.16)	1.89 (0.51)	< 0.05*	1.89 (0.21)	2.06 (0.20)	< 0.05*

*p < 0.05; **p < 0.01, Two-sample t-test for difference in means.

Table 4 shows the frequency distributions of the three forms and the results of the chi-square test for the North American and Turkish groups. In the former group, ovoid and tapered arch forms together made up more than 80% of the sample; but in the Turkish group, only 69% of the sample had ovoid and tapered arch forms. Square arch forms made up 30.3% of the Turkish group, but only 18.1% of the North American group. The most frequent arch forms seen were the ovoid arch form in the Turkish group and the tapered form in the North American group. The square arch form had the lowest frequency distribution in the Class I and Class II groups in both Turkish and North American groups; however, in the North American Class III samples, square arch forms were found at the highest frequency of 44% while ovoid arch forms were found at the highest frequency of 45% of the Class III samples in the Turkish.

**Table 4:** Distribution of arch forms by race and Angle classifications. Values presented as: percentage (n).

		White	Turkish	*p* value
Total	Square	18.1 (29)	30.3 (40)	0.006*
	Ovoid	38.1 (61)	42.4 (56)	
	Tapered	43.8 (70)	27.3 (36)	
Class I	Square	8.3 (5)	23.5 (8)	0.073 NS
	Ovoid	45.0 (27)	47.1 (16)	
	Tapered	46.7 (28)	29.4 (10)	
Class II	Square	4.0 (2)	27.6 (16)	0.002*
	Ovoid	36.0 (18)	37.9 (22)	
	Tapered	60.0 (30)	34.5 (20)	
Class III	Square	44.0 (22)	40.0 (16)	0.372 NS
	Ovoid	32.0 (16)	45.0 (18)	
Tapered	24.0 (12)	15.0 (6)

*p* > 0.05, NS = non-significant, *p < 0.01, Chi-square test.

Table 5 depicts arch dimension measurements and results of t-test obtained by regrouping the subjects into square, ovoid and tapered arch form samples. The Turkish had significantly narrower intercanine widths than the North American in the square arch form groups, and significantly larger intercanine widths than the North American in the ovoid and tapered arch form groups. The North American groups had significantly higher values for intermolar width in the square arch form and lower values for intermolar width in the tapered and ovoid arch form compared with the Turkish sample. Both ethnic groups showed significant increases in molar depth as the mandibular arches changed in form from square to ovoid to tapered.

**Table 5:** Comparison between North American and Turkish groups, by arch forms. Values presented as: Mean (SD).

	Square (n = 69)		p value	Ovoid (n = 117)		p value	Tapered (n = 106)		p value
	White (n = 29)	Turkish (n = 40)		White (n = 61)	Turkish (n = 56)		White (n = 70)	Turkish (n = 36)	
Intercanine width (mm)	29.96 (1.69)	29.23 (1.47)	< 0.05*	29.37 (1.34)	29.54 (1.68)	< 0.05*	28.44 (0.97)	28.60 (1.86)	> 0.05 NS
Intermolar width (mm)	52.24 (2.01)	50.18 (3.07)	< 0.05*	49.81 (2.27)	50.03 (2.69)	> 0.05	47.90 (1.95)	48.89 (2.12)	< 0.05*
Canine depth (mm)	5.26 (1.11)	5.49 (0.71)	< 0.05*	6.05 (0.76)	6.17 (0.99)	> 0.05	6.85 (1.06)	5.71 (1.08)	< 0.01**
Molar depth (mm)	26.16 (2.71)	24.69 (1.39)	< 0.05*	27.02 (1.78)	25.41 (1.98)	< 0.05*	27.52 (1.90)	26.07 (1.93)	< 0.05*
Canine W/D ratio	5.86 (0.92)	5.36 (0.70)	< 0.05*	4.91 (0.48)	4.73 (0.78)	> 0.05	4.24 (0.57)	4.73 (0.67)	< 0.05*
Molar W/D ratio	2.02 (0.19)	2.04 (0.20)	> 0.05	1.85 (0.12)	1.97 (0.16)	< 0.05*	1.75 (0.12)	1.88 (0.13)	< 0.05*

*p* > 0.05, NS = non-significant, *p < 0.05; **p < 0.01. ANOVA, 2-sample t-test.

## DISCUSSION

Some studies have reported on dental arch form, and a number of researchers have tried to establish the form unique to certain malocclusions, ethnic groups, and sex.^17^ ^,18^

Several classification schemes have been suggested, but the three basic arch forms that are commonly described by clinicians are tapered, ovoid and square arch forms.^19^ Clinically, it is important that arch form does not change during orthodontic treatment because occlusal stability depends on preservation of patient's original arch form.^3^ ^,20^

Preformed arch wires have been used frequently, although many reports have brought up the fact that application of the same arch wire in all cases can negatively affect post-treatment occlusal stability.^21^ ^,22^ Most manufacturers produce their arch wires based on North American or European arch forms; however, focusing on ethnic groups outside of these groups is more than a scholarly exercise. 

Several studies have described the shape of the dental arch by using different mathematical methods^23^ ^,^ ^24^ ^,^ ^25^ or by characterizing arch form through various measurements using the incisal edges and cusp tips as landmarks.^7^ ^,26^ These landmarks were used in studies carried out by Burris and Harris^27^ and Ling and Wong.^28^ Some researchers;^13^ ^,14,29,30^ however, used clinical bracket points as landmarks in their studies. Clinical bracket points corresponding to a bracket slot were used in this study, according to a method described in recent reports.^15^ ^,16,31,32^

These bracket points corresponded to bracket slot points that were mathematically estimated from the most facial portion of the proximal contact area of each tooth. Kook et al^13^ argued that using clinical bracket points as landmarks for measurement of dental arch shapes was of greater value for modern orthodontic treatment than the conventional incisal edge and cusp tip landmarks, since preformed superelastic arch wires are frequently used for clinical treatment.

The results of the current study clearly indicate that North American people have deeper arch forms in both canine and molar regions in comparison to the Turkish. Similar results were reported by both Gafni et al^29^ and Nojima et al.^14^ In the study by Nojima et al,^14^ the transverse widths of canines and molars were statistically significant larger for the Japanese than for the North American whites, and the ratio of anteroposterior lengths to canine and molar widths was also greater for the Japanese than for the North American whites. However, no statistically significant difference existed for the transverse widths of canines and molars between Turkish and white subjects. One can rank the total sample of Turkish mandibular arch dimension in relation to both North American whites and Japanese in the following order: North American whites < Turkish < Japanese. 

Braun et al^31^ stated, in their report on differences in arch dimensions between Angle classifications, that Class II mandibular arches exhibited generalized reduced arch width and depth compared with Class I arches, and that Class III mandibular arches had smaller arch depth and greater arch width than Class I arches. Our study showed that Class II canine and molar depth of the Turkish population was greater than in Class I and Class III subjects. This could be explained by the more tapered anterior curvature of Class II arches, which directly influences both canine and molar depth parameters. Class III arches showed significantly larger intercanine and molar widths than did the Class I and Class II arches in whites; this was consistent with previous reports.^13^ ^,14^ For both Turkish and North American whites, these findings also correlate with those of Nojima et al^14^ regarding Japanese subjects.

Felton et al^3^ reported little difference between the arch forms of Class I and Class II malocclusion groups. Our results showed that Class II arches for North American and Turkish groups were associated with a decreased frequency of the ovoid arch form and an increased frequency of the tapered arch form compared with Class I arches. For the Turkish group, similar results have been obtained in studies performed by Olmez et al.^11^

For Class III arches, the frequency of square arch form was the highest in all three groups, followed by ovoid and tapered arch forms. For both Turkish and North American whites, these findings also correlate with those of Nojima et al,^14^ regarding the Japanese sample. This can be similarity explained by the common developmental pattern of Class III malocclusion and the resultant dental compensation by lingual tipping of mandibular anterior teeth, which causes the anterior part of the mandibular arch to flatten. 

Table 5 shows significant difference between white and Turkish groups when comparing them only within each arch form type. Square arch forms had significant differences in size, except for molar W/D ratio. Ovoid arch forms showed significant differences in intercanine width, molar depth, and molar W/D ratio. Tapered arch forms were totally different in all areas, except for intercanine width.

Multiple studies have already reported differences in arch forms of subjects from various ethnic backgrounds.^13^ ^,14,29^

This study was the first comparison of arch forms between Turkish and North American white subjects. A study comparing the mandibular arches of Hispanic and Caucasian samples found that the square arch form was most prevalent in the Hispanic population (44%), followed by ovoid and tapering (28% each).^32^ Tapering arch form (44%) was more common in Caucasians, followed by ovoid (38%) and square (18%);^32^ thus, supporting that this anatomical guideline changes with race. Similar findings were reported by Nojima et al.^14^ The most frequent arch form was square in the Japanese group (45.6%), followed by ovoid (42.5%) and tapering (11.9%). A study on a Korean sample found ovoid (49.02%) to be the most frequent, followed by square (42.06%) and tapering (8.82%).^33^

The results of this study on a Turkish population, using a subjective method for arch form evaluation, reports that the most frequent arch form was ovoid (42%), followed by square (30%), with only 27% of tapered arch forms. 

Since there are differences in both arch dimension and frequency of arch form between Turkish and white subjects, as well as among malocclusion types, it is essential to select the best fit arch form for non-adaptable wires and to individualize the arch form in wires that can be altered in order to minimize round tripping of the dentition and to enhance stability of orthodontic results. 

## CONCLUSION

This study demonstrates that when treating Turkish patients, one should expect to use preformed ovoid arch form orthodontic wires in a significant percentage of patients. It is hoped that the arch form classification method will provide a practical guide in designing and fabricating preformed archwire forms for the Turkish population.
